# Operation Reliability Assessment for Cutting Tools by Applying a Proportional Covariate Model to Condition Monitoring Information

**DOI:** 10.3390/s121012964

**Published:** 2012-09-25

**Authors:** Gaigai Cai, Xuefeng Chen, Bing Li, Baojia Chen, Zhengjia He

**Affiliations:** State Key Laboratory for Manufacturing Systems Engineering, Xi'an Jiaotong University, Xi'an 710049, China; E-Mails: caigaigai.2008@stu.xjtu.edu.cn (G.C.); bli@mail.xjtu.edu.cn (B.L.); cbjia@163.com (B.C.); hzj@mail.xjtu.edu.cn (Z.H.)

**Keywords:** operation reliability assessment, condition monitoring information, distance evaluation technique, proportional covariate model, cutting tool

## Abstract

The reliability of cutting tools is critical to machining precision and production efficiency. The conventional statistic-based reliability assessment method aims at providing a general and overall estimation of reliability for a large population of identical units under given and fixed conditions. However, it has limited effectiveness in depicting the operational characteristics of a cutting tool. To overcome this limitation, this paper proposes an approach to assess the operation reliability of cutting tools. A proportional covariate model is introduced to construct the relationship between operation reliability and condition monitoring information. The wavelet packet transform and an improved distance evaluation technique are used to extract sensitive features from vibration signals, and a covariate function is constructed based on the proportional covariate model. Ultimately, the failure rate function of the cutting tool being assessed is calculated using the baseline covariate function obtained from a small sample of historical data. Experimental results and a comparative study show that the proposed method is effective for assessing the operation reliability of cutting tools.

## 1. Introduction

Tool failure is a major cause of unscheduled stoppages in the current manufacturing industry and is costly, not only in terms of time lost, but also in terms of capital destroyed [1]. Statistically, for modern machine tools, approximately 20% of downtime resulting in reduced productivity and economic losses is attributed to cutting tool failure [2]. The traditional tool replacement strategy based on regular time periods can reduce unplanned downtime and production losses to some extent, but tool degradation is a complex process and is easily affected by sophisticated and various machining environments [3]. There is a need to assess the real-time operation performance of a cutting tool to guarantee high machining quality and avoid unscheduled downtime to achieve higher economic efficiency and fewer disastrous accidents. For instance, during the manufacturing process of aircraft landing gear, the required processing precision is extremely high and scrapping a workpiece is not desirable because of the considerable cost. It is necessary to monitor the running states and assess the performance of cutting tools to create timely replacement policies or extend tool service life.

The past several decades have witnessed the rapid development of tool condition monitoring methods. As one of the outstanding examples in this field, wear-prediction-based monitoring methods have received great attention. Methods such as ANN [4], ANN ensemble [5], and SVM [6] have proved to be effective in tool condition monitoring [7]. Although these methods have played an important role in preventing degradation in machining quality, the wear value of a cutting tool is a single index; thus, it is difficult to provide a comprehensive estimation of the running condition of a cutting tool and the associated machining quality. It is necessary to find a method that can comprehensively assess the condition of a cutting tool and the performance of the corresponding machining process. Reliability theory describes the probability that a system will complete its expected function during an interval of time. The reliability function obtained from the reliability assessment of a cutting tool could provide a more detailed analysis of the cutting tool's performance [8]. Therefore, an effective reliability assessment of a cutting tool is necessary to develop a more effective real-time tool replacement strategy, avoid unplanned shutdowns, guarantee high machining quality, and promote productivity.

Considerable efforts have been made by researchers and engineers to investigate the reliability of cutting tools. Hitomi *et al.* derived the reliability of cutting tools based on the tool-wear distribution estimated from tool-wear experiments [9]. To quantify the reliability of carbide tools, Negishi and Aoki studied the influence of feed rate on the life of cutting tools during intermittent cutting [10]. Liu and Makis used a proportional hazard model to assess the reliability of cutting tools in variable conditions by taking machining conditions into consideration [11]. Based on the theorem of total probability, Klim *et al.* obtained the mean time to failure of cutting tools from the reliability function through a stochastic model [8]. Wang *et al.* predicted the reliability of cutting tools based on a reliability-dependent failure rate model that involves two decay factors: the embedded decay factor and the process-dependent decay factor [12]. Lin *et al.* used the normal distribution model to calculate the reliability of cutting tools in high-speed turning and revealed that the tool flank wear rate can be described by the reliability degradation rate [13]. Hsu *et al.* proposed a non-homogenous continuous-time Markov process to model the tool wear process and performed a reliability assessment of a cutting tool with multi-state deterioration [14].

The investigations mentioned above have made significant progress in cutting tool reliability analysis. However, some difficult problems still remain. Most of the above studies, to some extent, rely on the complex mechanisms of tool wear or machining conditions, which are generally unstable. More importantly, these studies only provide a general overall estimation of a group of cutting tools. The estimation is useful to manufacturers that produce units in high volumes [15]. However, the aforementioned approaches become less powerful and are not accurate for the operation reliability assessment of cutting tools during field use. Unfortunately, engineers are particularly concerned about such characteristics of operation performance and operation reliability. To evaluate operation reliability that can effectively reflect the performance of a given cutting tool, it is preferable to develop a reliability assessment approach that depends less on professional knowledge of the complex wear mechanisms of the cutting tool and machining environment.

Accurate condition monitoring information of a cutting tool, including direct and indirect sensor-based information, can effectively reflect the real-time running state of the cutting tool. Over the past decades, a considerable number of studies have been carried out to focus on tool condition monitoring by using direct [16,17] or indirect sensor-based methods. Owing to the advantages of a less complicated setup and less reliance on professional knowledge of complex wear mechanisms, indirect sensor-based approaches have been widely preferred over direct sensor-based methods [18]. Among all of the indirect sensor-based information that can be obtained, such as cutting forces [19], vibration signals [20–23], acoustic emission signals [23–26], current signals [27] and internal CNC signals [28], vibration signals have been widely used in equipment condition monitoring for their advantages of low price, easy implementation and continuous on-line testing [29]. Investigation results show that the features extracted from vibration signals in the time domain or the frequency domain are sensitive to tool wear [30,31] and insensitive to the variation in cutting conditions [32]. It is beneficial to take advantage of real-time sensor-based vibration signals to evaluate the operation reliability of a cutting tool. However, there still remain some challenges in establishing an appropriate reliability assessment model to represent the relationship between vibration signals and operation reliability.

This study proposes an operation reliability assessment approach for cutting tools by applying the proportional covariate model (PCM) to construct the relationship between condition monitoring information and operation reliability. In the proposed method, wavelet packet transform (WPT) is utilised to analyse vibration signals. In this method, a feature set consists of the wavelet packet energy of each frequency-band signal and the wavelet packet energy entropy is obtained. An improved distance evaluation technique is performed to select sensitive features associated with cutting tool degradation and to determine the weights of sensitive features. Then, the feature covariate function of the sensitive features is constructed. Subsequently, the baseline covariate function is quantitatively obtained by integrating lifetime data and sensitive features extracted from historical vibration signals. The PCM is established to calculate the failure rate function of the cutting tool in operation. Finally, the operation reliability of the cutting tool during processing is assessed via the failure rate function. Experiments on a CNC lathe were carried out to verify the effectiveness of the proposed method. The assessment result verified that the presented approach is capable of and practical for evaluating the operation reliability of a given cutting tool during processing.

The rest of the paper is organised as follows: Section 2 introduces the proposed operation reliability assessment approach. Section 3 describes the experimental setup and sensor-based information acquisition. In Section 4, the proposed method is verified and the main results are discussed. Finally, conclusions are drawn in Section 5.

## 2. Operation Reliability Assessment Approach for Cutting Tools

The key to the proposed operation reliability assessment approach is to establish the relationship between the condition monitoring information and the operation reliability of a cutting tool by applying PCM. Section 2.1 describes the rationale of the PCM. The calculation methods of the two key functions (the feature covariate function and the baseline covariate function) for constructing PCM are presented in Section 2.2 and Section 2.3, respectively. The algorithm of the proposed method is illustrated in Section 2.4.

### 2.1. Proportional Covariate Model

It is commonly understood that the deterioration of a mechanical system generally tends to increase the probability of failure. Accurate condition monitoring information of a system can reflect its deterioration progress. It is reasonable to assume that features extracted from condition monitoring information or a function of these features is proportional to the failure rate of the system. This assumption has been widely used to study mechanical systems and has been verified by Sun [33,34]. PCM was proposed to estimate the failure rate of a mechanical system by using condition monitoring information based on this assumption [33]. PCM was constructed to forge a relationship between the failure rate function and condition monitoring information.

Suppose that at time *t*, **Z**(*t*) = (*Z_r_*_1_(*t*), *Z_r_*_2_(*t*), …, *Z_rM_*(*t*))^T^ is an *M*-dimensional signal feature set extracted from the condition monitoring information of a system. *ψ*(**Z**(*t*)) is the feature covariate function of the feature set **Z**(*t*). It is time dependent and represents the running states of the system. *h*(*t*) is the failure rate function of the operation reliability. PCM is formulated as follows [33]:

(1) $$\psi (\text{Z}(t))=c_{0}(t)h(t)$$

where *c*_0_(*t*) represents the proportional relationship between the failure rate function and the condition monitoring information. Thus, the failure rate function can be obtained as:

(2) $$h(t)=\frac{\psi (\text{Z}(t))}{c_{0}(t)}$$

There are two key techniques used to estimate the failure rate function: one is the construction of the feature covariate function *ψ*(**Z**(*t*)), and the other is the creation of the baseline covariate function *c*_0_(*t*).

Constructing a suitable mathematical model for *ψ*(**Z**(*t*)) plays a critical role in improving the accuracy of failure rate estimation, especially when *M* > 1. In this study, **Z**(*t*) is an *M*-dimensional sensitive feature set extracted and selected from a vibration signal. Many statistical models are available for the formulation of *ψ*(**Z**(*t*)) [35,36]. The exponential model, one of the most commonly used in practice, is employed in this paper as follows:

(3) $$\psi \left(\text{Z}(t)\right)=\exp(\sum_{m=1}^{M}w_{m}Z_{\text{rm}}(t))=\exp({\text{w}}^{\text{T}}\text{Z}(t))$$

where *Z*(*t*) = (*Z_r_*_1_(*t*), *Z_r_*_2_(*t*), …, *Z_rM_*(*t*))^T^ is the sensitive feature set and the variables in **w** = (*w*_1_, *w*_2_, …, *w_M_*)^T^ are the corresponding feature weights. The detailed construction method for *ψ*(**Z**(*t*)) is described in Section 2.2.

Furthermore, studies have shown that there are two approaches to estimate the baseline covariate function: (1) Estimate the function from historical failure data and condition monitoring information. (2) In the case of sparse or even zero historical data, the baseline covariate function can also be determined according to the anecdotal experience of operators of plants and/or by using supplementary information such as data from accelerated life tests. In this study, we focus on the first approach. The baseline covariate function is quantitatively calculated by using historical failure data and condition monitoring information. The detailed approach is presented in Section 2.3.

Once both the feature covariate function *ψ*(**Z**(*t*)) and the baseline covariate function *c*_0_(*t*) are determined, the failure rate function of the cutting tool during processing can be calculated by using the condition monitoring information of the cutting tool as follows:

(4) $$\tilde{h}(t)=\frac{\psi (\tilde{\text{Z}}(t))}{c_{0}(t)}=\frac{\exp({\text{w}}^{\text{T}}\tilde{\text{Z}}(t))}{c_{0}(t)}$$

where **Z̃**(*t*) = (*Z̃_r_*_1_(*t*), *Z̃_r_*_2_(*t*), …, *Z̃_rM_*(*t*))^T^ is the sensitive feature set of the cutting tool that is being assessed.

It has been stated that PCM was developed to estimate the failure rate of a system to ultimately perform a reliability assessment of a cutting tool. Some of the most essential definitions of reliability theory are reviewed as follows. The failure rate *h*(*t*) is defined as the ratio of probability density function *f*(*t*) to reliability *R*(*t*):

(5) h(t)=f(t)/R(t)

where *f*(*t*) is defined as:

(6) $$f(t)=\frac{\text{d}F(t)}{\text{d}t}=-\frac{\text{d}R(t)}{\text{d}t}$$

yielding:

(7) $$R(t)=\exp(-\int_{0}^{t}h(t)\text{d}t)$$

Eventually, the reliability assessment of a cutting tool can be obtained by PCM.

### 2.2. The Construction of the Feature Covariate Function

To construct the feature covariate function, a two-stage feature covariate function construction approach based on WPT and an improved distance evaluation technique is presented in this subsection. Section 2.2.1 details the first stage: feature extraction by WPT. Section 2.2.2 presents the second stage: feature selection and weighting by using the improved distance evaluation technique.

#### 2.2.1. Feature Extraction

Feature extraction is critical to acquiring characteristic information regarding cutting tool degradation. It has been reported that the characteristics mainly focus on some specific frequency bands [22]. Therefore, WPT is performed to decompose the vibration signal into a set of distinct frequency bands.

Wavelet transform (WT) is a powerful multi-resolution analysis method that is localised both in the time domain and the frequency domain [37–39]. However, an unavoidable drawback of WT is that the frequency resolution in the high-frequency region is rather poor [40]. As a generalisation of WT, WPT further decomposes the high-frequency bands and thus generates a finer frequency-band partition over the whole analysed frequency interval [40,41]. In this study, WPT is utilised to analyse the vibration signals of a cutting tool.

Study [29] shows that the variations in the wavelet packet energies within some frequency bands are consistent with cutting tool degradation. Moreover, this can be easily recognised regardless of the cutting parameters. The energy value of each frequency band can effectively reflect the running condition of a cutting tool and provide useful information to conduct a cutting tool operation reliability assessment. Thus, single branch reconstruction is performed, and the wavelet packet energy of each frequency band is then calculated. The benefits of single branch reconstruction are manifold, including the preservation of the analysis resolution in both the time domain and the frequency domain, as well as the suppression of frequency aliasing.

Let *X_(m,n)_* represent the *n*th frequency band signal of the *m*th level decomposition and *S_m,n_* represent the single branch reconstruction of *X_(m,n)_*; the corresponding wavelet packet energy *E_m,n_* is calculated as follows [42]:

(8) $$E_{m,n}=\int {\left|S_{m,n}(t)\right|}^{2}\text{d}t=\sum_{i=1}^{L}{\left|r_{m,n}(i)\right|}^{2}$$

where *r_m,n_* (*m* = 1,2, …, *N;n* = 1,2, …, 2*^m^*) is the amplitude of the reconstructed signal *S_m,n_*, and *L* represents the length of the signal. For the generalisation of the application, the normalised wavelet packet energy of *S_m,n_* is calculated as follows:

(9) $$P_{m,n}=\frac{E_{m,n}}{\sum_{n}E_{m,n}}$$

Energy entropy is capable of detecting the change in signal energy in different frequency bands and reveals the amount of information stored in the observed signal. The wavelet packet energy entropy quantifies the statistical properties of the instantaneous power of a vibration signal that are largely unaffected by changes in the machining environment. The wavelet packet energy entropy *E_m_* is defined as:

(10) $$E_{m}=-\sum_{n}P_{m,n}\log P_{m,n}$$

Thus, the original signal feature set of the vibration signal is constructed as follows:

(11) $$K=\{P_{m,1},P_{m,2},\cdots ,P_{m,2^{m}},E_{m}\}$$

#### 2.2.2. Feature Selection and Weighting

The extracted features in the original feature set have various degrees of importance in reflecting the degradation severity of a cutting tool from different aspects. Some features are sensitive and closely related to the degradation of the cutting tool, while others are not. Irrelevant or redundant features not only mask information that is useful for an operation reliability assessment but also increase the computational burden. Hence, an improved distance evaluation technique is introduced to select sensitive features of cutting tool degradation and reduce irrelevant or redundant features from the original feature set. The distance evaluation technique is carried out based on the “intra-class” and “inter-class” distances [43]. In other words, the features that engender longer intra-class distances and shorter inter-class distances are regarded as superior. However, the distance evaluation technique ignores the difference between the aggregation degree among conditions and the intra-class distance. The improved distance evaluation technique enhances the evaluation results defining and calculating a compensation factor.

Suppose that a feature set of *C* conditions is:

(12) $$\left\{K_{m,c,j},m=1,2,\cdots ,M_{c};c=1,2,\cdots ,C;j=1,2,\cdots ,J\right\}$$

where *K_m,c,j_* is the *j*th feature of the *m*th sample under the *c*th condition, *M_c_* is the sample number of the *c*th condition, and *J* is the feature number of each condition. The feature selection steps based on the improved distance evaluation technique are as follows.

- Calculate the average distance of the same condition samples:
  (13) $$d_{c,j}=\frac{1}{M_{c}\times (M_{c}-1)}\sum_{i,m=1}^{M_{c}}\left|K_{m,c,j}-K_{i,c,j}\right|,i,m=1,2,\cdots ,M_{c},i\neq m$$
  then determine the average intra-class distance of *C* conditions $d_{j}^{\left(w\right)}=\frac{1}{C}\sum_{c=1}^{C}d_{c,j}$.
- Define and calculate the intra-class difference factor of $d_{j}^{\left(w\right)}$ as follows:
  (14) $$v_{j}^{\left(w\right)}=\max(d_{c,j})/\min(d_{c,j})$$
- Compute the average feature value of all samples under the same condition:
  (15) $$u_{c,j}=\frac{1}{M_{c}}\sum_{m=1}^{M}K_{m,c,j}$$
  then determine the average distance between different sample conditions:
  (16) $$d_{j}^{\left(b\right)}=\frac{1}{C\times (C-1)}\sum_{c,e=1}^{C}\left|u_{e,j}-u_{c,j}\right|,c,e=1,2,\cdots ,C,c\neq e$$
- Compute the inter-class difference factor:
  (17) $$v_{j}^{\left(b\right)}=\frac{\max(\left|u_{e,j}-u_{c,j}\right|)}{\min(\left|u_{e,j}-u_{c,j}\right|)},c,e=1,2,\cdots ,C,c\neq e$$
- Define and calculate the compensation factor:
  (18) $$\lambda_{j}={\left[\frac{v_{j}^{\left(w\right)}}{\max(v_{j}^{\left(w\right)})}+\frac{v_{j}^{\left(b\right)}}{\max(v_{j}^{\left(b\right)})}\right]}^{-1}$$
- Calculate the ratio of $d_{j}^{\left(b\right)}$ to $d_{j}^{\left(w\right)}$ and create the compensation factor:
  (19) $$a_{j}=\lambda_{j}\frac{d_{j}^{\left(b\right)}}{d_{j}^{\left(w\right)}}$$

Finally, normalise the value of *a_j_* and use its maximum value to determine the distance evaluation criterion:

(20) $${\bar{a}}_{j}=\frac{a_{j}}{\max\{a_{j};j=1,2,\cdots ,J\}}$$

Obviously, high a value of *ā_j_* indicates that the corresponding feature is quite capable of distinguishing different degradation conditions of a cutting tool. By setting a threshold value *ϕ* ∈ [0,1], the first *M* most sensitive features can be selected from the original feature set according to the criterion *ā_j_* ≥ *ϕ*.

Although sensitive features have been selected from the original feature set via the improved distance evaluation technique, the selected features have different sensitivities in reflecting the running condition of a cutting tool. Feature weighting is implemented here to achieve a more dependable reliability assessment result. The basic idea of feature weighting is to multiply each feature by a number within the interval [0,1] that is proportional to the capability of a given feature to distinguish between different conditions. Fortunately, the value of the distance evaluation criterion represents a feature's sensitivity to different conditions. Thus, it is rational to use the acquired distance evaluation criteria as weight factors of the sensitive features, that is:

(21) $$w_{m}={\bar{a}}_{m}$$

As mentioned in Section 2.1, the recommended form of the feature covariate function *ψ*(**Z**(*t*)) is as expressed in Equation (3) in the case of multiple features. The determined distance evaluation criteria act as the feature weights of each sensitive feature and are then substituted into Equation (3). Thus, the feature covariate function *ψ*(**Z**(*t*)) can be fully constructed.

### 2.3. Quantitative Calculation of the Baseline Covariate Function

As previously mentioned in Section 2.1, there are two approaches to estimate the baseline covariate function. In this paper, the first approach is employed. According to Equation (2), a set of discrete values of *c*_0_(*t*) can be obtained:

(22) $$c_{0}(t_{i})=\frac{\psi (\text{Z}(t_{i}))}{h_{in}(t_{i})}=\frac{\exp({\text{w}}^{\text{T}}\text{Z}(t_{i}))}{h_{in}(t_{i})}(i=1,2,\cdots ,m_{c})$$

where *ψ*(**Z**(*t_i_*)) is the discrete value of the feature covariate function calculated by historical condition monitoring information, *m_c_* is the sample size of historical vibration signals, and *h_in_*(*t_i_*) is the initial failure rate of the condition monitoring information at the corresponding moment. *ψ*(**Z**(*t_i_*)) is determined by historical condition vibration signals according to Section 2.2.

To evaluate the initial failure rate function *h_in_*(*t*), a proper failure distribution for the cutting tool being analysed must first be determined. A Weibull distribution is used to describe the tool failure [13,37]. The failure rate function of a Weibull distribution with two parameters is:

(23) $$h(t)=(\beta /\eta )({t/\eta )}^{\beta -1}$$

where *β* and *η* are the shape parameter and the scale parameter of the Weibull distribution, respectively.

To make sure that the failure distribution of the cutting tool obeys a Weibull distribution, the hypothesis test is implemented. The failure distribution of the cutting tool is identified based on the historical lifetime data {*τ_n_* : *n* = 1,2,…,*m_f_*} by the hypothesis test, where *m_f_* is the number of lifetime data points. Once the failure distribution is determined, maximum likelihood estimation (MLE) is employed to identify the unknown parameters *β* and *η* by using the historical lifetime data.

By utilising the initial failure rate function *h_in_*(*t*) and the historical feature covariate function *ψ*(**Z**(*t*)), a set of discrete values of *c*_0_(*t_i_*) (*i* = 1,2,…,*m_c_*) are obtained by Equation (22). According to the recommendation made in [33], a multiplicative model is chosen to represent the baseline covariate function, that is:

(24) $$c_{0}(t)=at^{b}$$

Then, the baseline covariate function can be estimated from the discrete data set {*t_i_*, *c*_0_(*t_i_*) : *i* = 1,2,…,*m_c_*}.

### 2.4. The Algorithm of the Operation Reliability Assessment Approach

**Figure 1.**
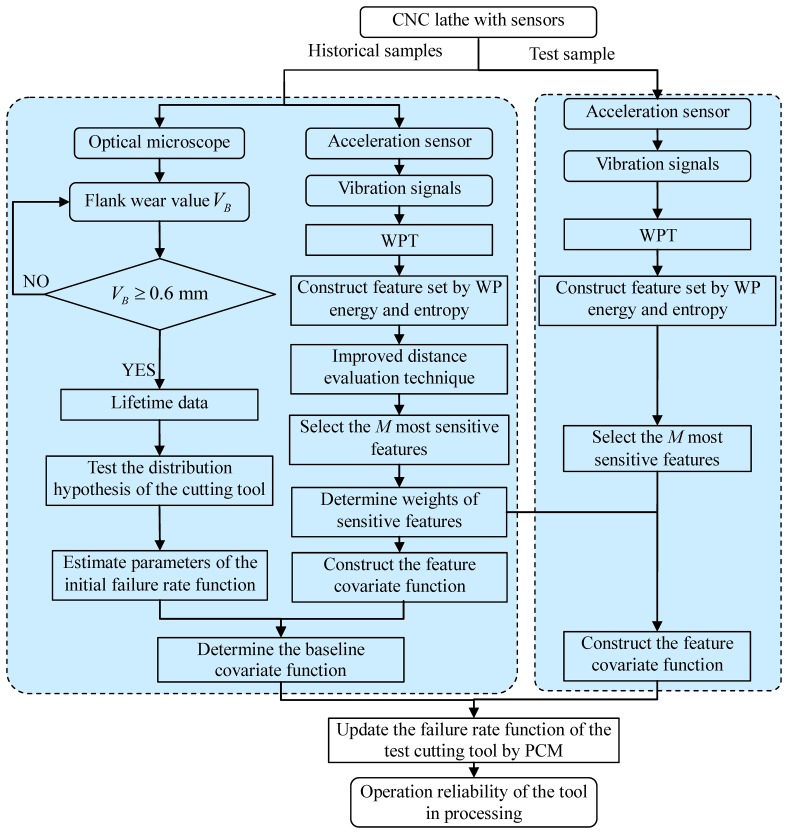
Flowchart of the proposed method.

To assess the operation reliability of the cutting tool that is to be assessed, a sensor-based data acquisition process that can record useful information about the cutting tool must be carried out first. In the present study, the vibration signals of the cutting tool and optical microscopy-based flank wear values are recorded. The lifetimes of the historical samples are determined under the condition that their flank wear value *V_B_* exceeds 0.6 mm according to ISO3685. Then, the proposed approach is applied to estimate the operation reliability of the cutting tool to be assessed. The flowchart of the method is depicted in Figure 1. The detailed steps are summarised as follows:

*Step 1:* (*Determine proper failure distribution for the cutting tool*): Identify the failure distribution of the cutting tool by implementing a hypothesis test using the historical lifetime data {*τ_n_* : *n* = 1,2,…,*m_f_*}.
*Step 2:* (*Estimate the initial failure rate function*): Once the failure distribution is determined, MLE is adopted to evaluate the parameters of the initial failure rate function *h_in_*(*t*) by using historical lifetime data.
*Step 3:* (*Feature extraction*): To acquire characteristic information regarding cutting tool degradation, WPT is performed to extract features from the vibration signals.
*Step 4:* (*Feature selection and weighting*): Feature selection is performed through the improved distance evaluation technique. And the distance evaluation criteria of each sensitive feature act as feature weights to construct the feature covariate function.
*Step 5:* (*Construct the feature covariate function*): The feature covariate function is constructed using the feature weights of each sensitive feature based on step 3 and step 4.
*Step 6:* (*Calculate the baseline covariate function*): After the initial failure rate function and the feature covariate function are obtained using information from the historical samples, discrete baseline covariate values are calculated, and the baseline covariate function is then calculated by a regression analysis technique using the discrete baseline covariate values.
*Step 7:* (*Update the failure rate function of the test cutting tool*): To update the failure rate function of the test cutting tool, the vibration signals of the test cutting tool are monitored by an acceleration sensor. The feature covariate function of the cutting tool is constructed according to step 3 and step 4.
  Then, PCM is constructed to update the failure rate function of the test cutting tool based on the feature covariate function and the baseline covariate function of the cutting tool:
  (25) $$\tilde{h}(t_{j})=\frac{\exp(\sum_{m=1}^{M}w_{m}{\tilde{Z}}_{rm}(t_{j}))}{c_{0}(t_{j})}=\frac{\exp({\text{w}}^{\text{T}}\tilde{\text{Z}}(t))}{c_{0}(t_{j})}(j=1,2,\cdots ,m_{n})$$
  Assuming that the failure rate function of the cutting tool is of the form *h*(*t*) = (*β*/*η*)(*t*/*η*)^*β*−1^ shown in Equation (23), *h̃*(*t*) can be evaluated using {*t_j_*, *h̃*(*t_j_*)} by regression analysis. Thus, the updated failure rate function of the test cutting tool is obtained.
*Step 8:* (*Assess the operation reliability*): Calculate the operation reliability of test cutting tool that is to be assessed using the updated failure rate function via Equation (7).

## 3. Experimental Setup and Sensor-Based Information Acquisition

**Figure 2.**
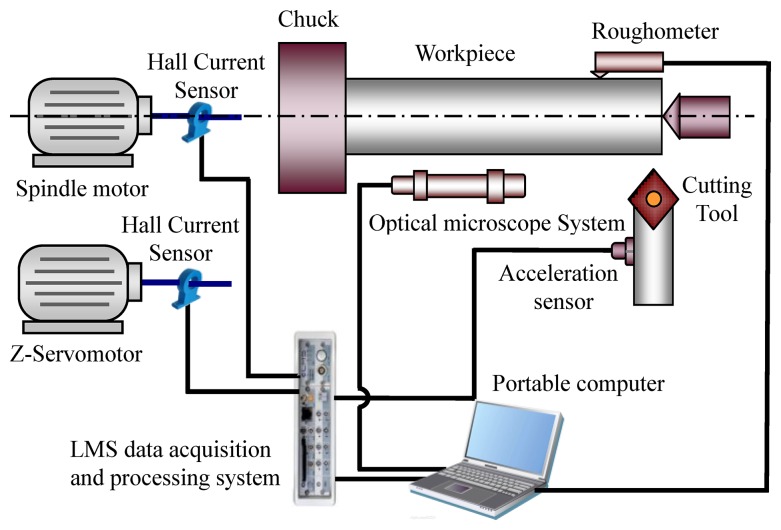
The schematic diagram of the experimental setups.

**Figure 3.**
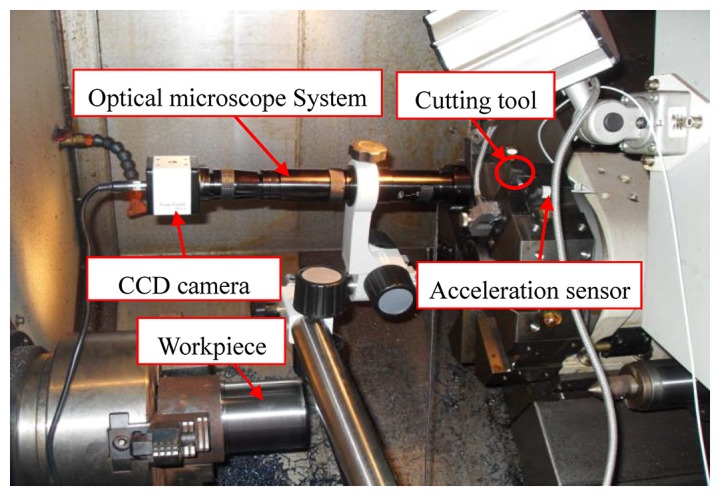
Experimental system and sensor locations.

To test the effectiveness of the proposed operation reliability assessment method, an experimental system for the studied cutting tool was designed and carried out on a CNC lathe. Figure 2 shows a schematic diagram of the experimental setup. Figure 3 shows the experimental system of the test rig and the locations of sensors. In the experiment, the carbide cutting tool was utilised to process 45# steel bars. The vibration signals of the cutting tool were monitored by an acceleration sensor, sent to a data acquisition and signal processing system (LMS SCADAS305), and finally stored in a portable computer. The flank wear value *V_B_* of the cutting tool was measured by an optical microscopy system with a CCD camera, an adjustable LED annular source, and a micrometer. The surface roughness data of the workpiece, the current signals of the spindle motor and the Z-servo motor were also monitored for further study. Table 1 shows the experimental conditions. Table 2 lists the detailed information obtained by the sensors used in the experiment.

To accumulate sufficient vibration signals and lifetime data from the cutting tool, vibration signals and flank wear values were monitored during the cutting process under constant condition. Vibration signals were sampled every 2 min during the cutting process with a sampling frequency of 32,768 Hz and a sample interval of 2 s.

## 4. Results and Discussion

**Figure 4.**
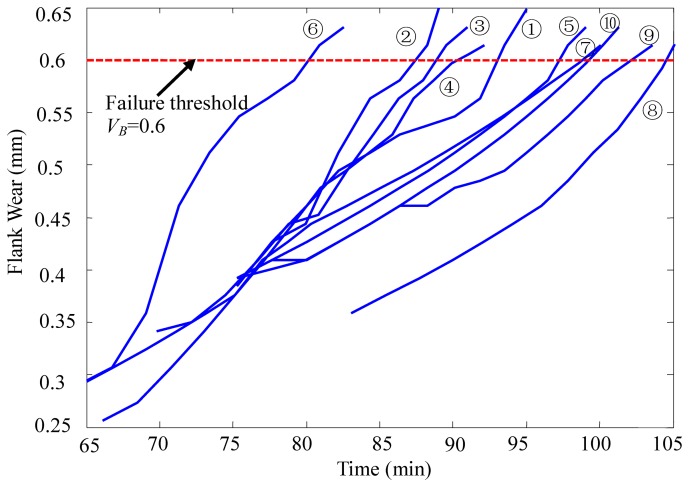
Variation curves of the cutting tools flank wear values.

First, 10 cutting tools were investigated as historical samples. The variation curves of the cutting tools' flank wear values are shown in Figure 4. The cutting tool is considered to have failed when the flank wear value *V_B_* ≥ 0.6 mm. Lifetime data of all 10 cutting tools were obtained according to the measured flank wear values of the cutting tools.

### 4.1. Initial Failure Distribution Determination

**Figure 5.**
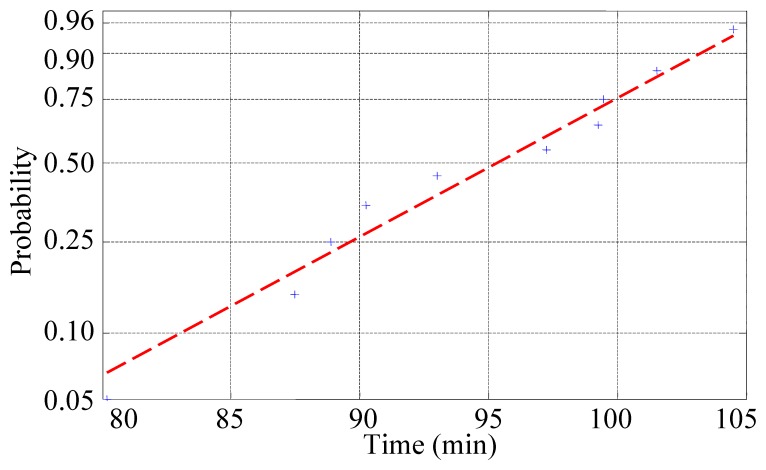
Weibull distribution fitting test.

The hypothesis test was implemented to analyse the failure distribution of the cutting tool. The result is shown in Figure 5. It is rational to assume that the failure distribution of the cutting tool obeys a Weibull distribution. Then, the failure distribution of the cutting tool was estimated by MLE:

$$F\text{(}t\text{)}=1-\exp\left[-{\left(\frac{t}{97.358}\right)}^{15.896}\right]$$

The initial failure rate function of the cutting tool is $h_{in}\text{(}t\text{)}=\frac{15.896}{97.358}{\left(\frac{t}{97.358}\right)}^{14.896}$.

### 4.2. Construction of the Feature Covariate Function

The vibration signals of the 10 historical samples were investigated by WPT and the improved distance evaluation technique to construct the feature covariate function.

#### 4.2.1. Feature Extraction and Selection

**Figure 6.**
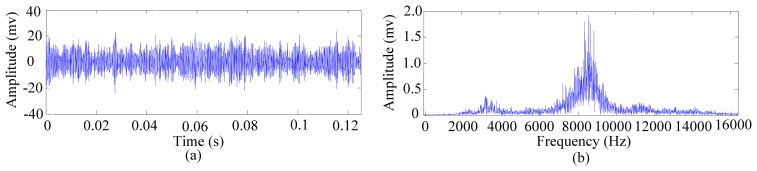
(**a**) Vibration signal and (**b**) spectrum of tool No. 3 after running for 69.5 min.

**Figure 7.**
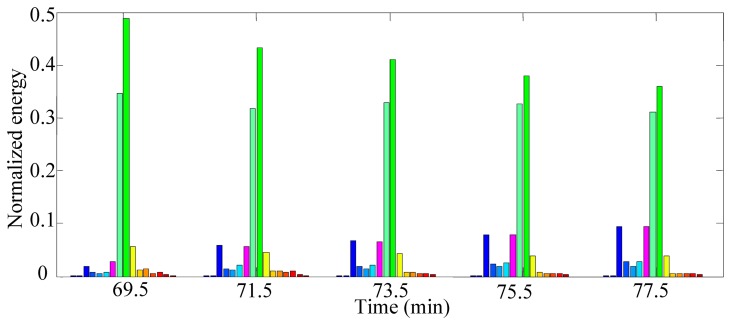
Changes in WPT energy spectrum.

To extract the features sensitive to cutting tool wear, WPT was adopted to decompose the vibration signals of the cutting tool. According to the characteristics of the vibration signals, the mother wavelet used here should have the properties of orthogonality, short support, symmetry and a certain order of vanishing moment. Thus, the Daubechies 10 (db10) wavelet was chosen as the mother wavelet to analyse the vibration signals of the cutting tool. The decomposition level can be determined by the sampling frequency and frequency scope of the concentrated energy of the signal obtained from spectrum analysis [44]. First, the vibration signals were analysed to construct the feature covariate function. Tool No. 3 was taken as an example to demonstrate the procedure of feature extraction and feature set construction. Figure 6 shows the raw vibration signal of the tool after running for 69.5 min and its spectrum. The energy of the signal is mainly concentrated in two specific frequency bands, [2,4] KHz and [7,10] KHz, the latter of which contains richer energy information. According to the concentration of the energy shown in Figure 6(b), the vibrations signal was decomposed into four levels by WPT. The wavelet packet energies of the sixteen frequency-band signals and the WPT energy entropy of the vibration signal were calculated according to Equation (9) and Equation (10), respectively. Then, the original feature set *K* = {*P*_4,1_,*P*_4,2_,…,*P*_4,16_,*E*_4_} of the vibration signal was constructed at each sample time. Figure 7 displays the variation of the normalised wavelet packet energy values of tool No. 3 running from 69.5 min to 77.5 min. It can be observed that the frequency-band energy distribution presents certain variation regularity during the manufacturing process. For instance, the normalised energy values of band 9 decrease from 0.487 to 0.360 with the degradation of the cutting tool. The above analysis procedures were also applied to the other nine cutting tools, and signal feature sets at different running times were obtained with similar vibration regularities.

**Figure 8.**
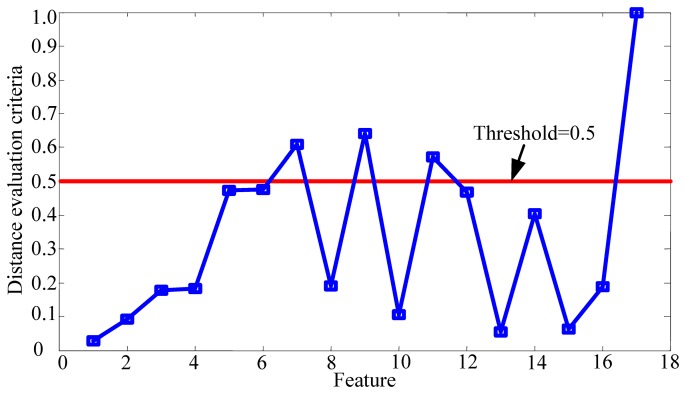
Distance evaluation criteria of all features.

The wear states of the cutting tool were divided into three different conditions according to the tested flank wear values [14,45]. Each feature set obtained from the vibration signal of the cutting tool corresponds to a certain condition. Then, based on the acquired corresponding feature sets of each condition, the improved distance evaluation technique was performed to evaluate the ability of the feature to distinguish different conditions. Figure 8 displays the distance evaluation criteria *ā_j_* (*j* = 1,2,…, 17) of all of the features. A high *ā_j_* value means that the corresponding feature is highly sensitive to degradation. To select the most sensitive features from the original feature set and keep only the important features, a threshold value *ϕ* must be properly selected. According to [43], the range median of the evaluation criteria, 0.5, was chosen as the threshold to select the most sensitive features in the study. Therefore, the four most sensitive features (*P*_4,7_,*P*_4,9_, *P*_4,11_,*E*_4_) were selected. The corresponding distance evaluation criteria of the selected features are **w** = (0.6085, 0.6406, 0.5717, 1)^T^.

#### 4.2.2. The Construction of the Feature Covariate Function

After the four most sensitive features were selected from the original feature set, the distance evaluation criteria of the four selected features acted as weight factors to construct the feature covariate function. The corresponding feature weights set for the selected sensitive features set **Z**(*t*) = (*P*_4,7_(*t*), *P*_4,9_(*t*), *P*_4,11_(*t*),*E*_4_(*t*))^T^ was **w** = (0.6805, 0.6406, 0.5717, 1)^T^. Then, the feature covariate function of the sensitive features was constructed:

$$\psi (\text{Z}(t))=\exp({\text{w}}^{\text{T}}\text{Z}(t))=\exp(0.6085P_{4,7}(t)+0.6406P_{4,9}(t)+0.5717P_{4,11}(t)+E_{4}(t))$$

### 4.3. The Quantitative Calculation of the Baseline Covariate Function

According to Section 2.3, discrete values of the baseline covariate function can be obtained based on the initial failure rate function and the constructed feature covariate function of the 10 historical cutting tools. Then, the baseline covariate function can be estimated by using regression analysis based on the discrete values. The estimation result of the baseline covariate function for the cutting tool is:

$$c_{0}(t)=1.343\times 10^{27}t^{-12.71}$$

### 4.4. Failure Rate Function Update and Operation Reliability Assessment Using PCM

To assess the operation reliability of tool No. 11 during operation, the feature covariate function of tool No. 11 was constructed by using the corresponding vibration signals. Then, the PCM was utilised to update the failure rate function of tool No.11. According to Equation (25), discrete failure rates for the tool can be obtained as follows:

$$\tilde{h}(t_{j})=\frac{\psi (\tilde{\text{Z}}(t_{j}))}{c_{0}(t_{j})}=\frac{\exp(0.6085{\tilde{P}}_{4,7}(t_{j})+0.6406{\tilde{P}}_{4,9}(t_{j})+0.5717{\tilde{P}}_{4,11}(t_{j})+{\tilde{E}}_{4}(t_{j}))}{1.343\times 10^{27}t_{j}^{-12.71}}(j=1,2,\cdots ,m_{n})$$

where *m_n_* = 16 is the number of sensitive feature sets extracted from the vibration signals of tool No. 11. Consequently, the failure rate function for tool No. 11 was acquired by regression analysis, that is:

$$\tilde{h}(t)=\frac{14.03}{98.79}{\left(\frac{t}{98.79}\right)}^{13.03}$$

**Figure 9.**
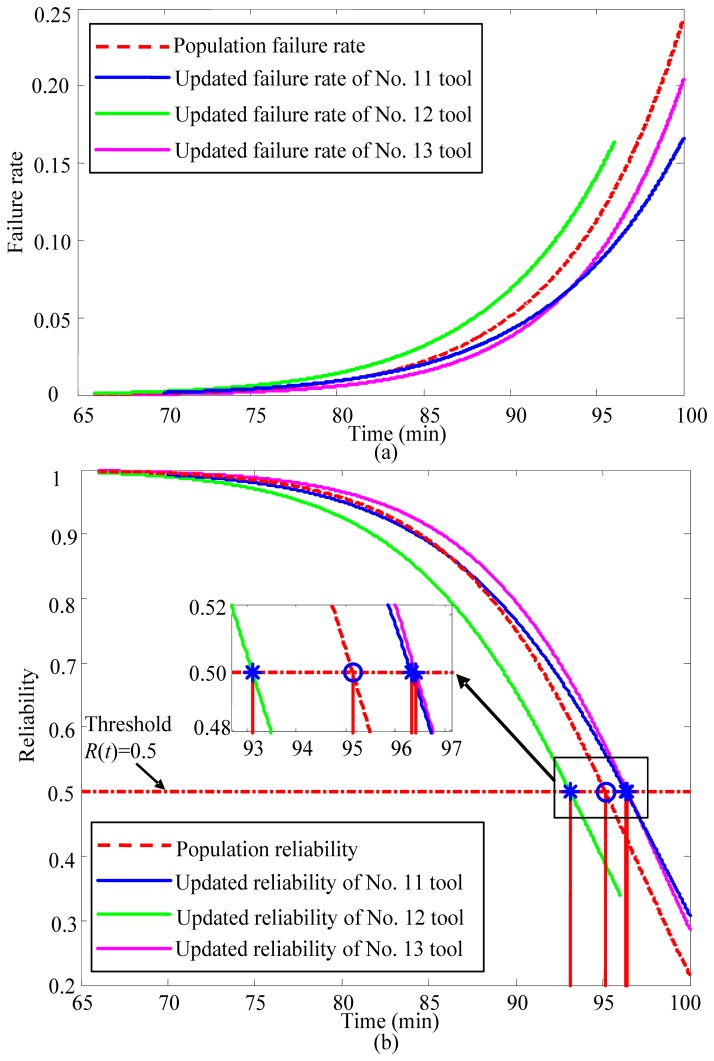
(**a**) Failure rate comparison. (**b**) Reliability comparison.

Finally, the operation reliability of the cutting tool can be calculated according to Equation (7). As mentioned in the Introduction, the conventional reliability assessment method fails to reflect the characteristics of a specific cutting tool. To verify that the proposed method can overcome this drawback, the updated failure rate of tool No. 11 was compared with the population failure rate calculated by the conventional reliability assessment method in Figure 9(a). The reliability assessment result of the proposed method was compared with the population reliability in Figure 9(b).

To test the effectiveness of the reliability assessment result, the lifetime of the cutting tool is estimated based on the reliability assessment of the proposed method and compared with the measured real lifetime of the tool by experiment. Based on engineering practice, in this paper, *R*(*t*) = 0.5 is set to be the failure threshold of the cutting tool. The estimated lifetime of tool No. 11 is 96.33 min based on the proposed method. Meanwhile, according to the conventional reliability assessment method, the estimated lifetime of tool No. 11 is 95.13 min. The experimentally measured real lifetime of the cutting tool is 96.83 min (*V_B_* = 0.6 mm). Clearly, the proposed method provides a more accurate reliability and lifetime evaluation for the cutting tool. On the other hand, based on the assessment of the proposed method, the operation reliability of the cutting tool is 0.47 when the cutting tool is running for 96.83 min, which is also consistent with the cutting tool's actual running state.

To further confirm the effectiveness and general applicability of the proposed method, it was used to assess the failure rate and operation reliability of another two cutting tools. The assessment results are also shown in Figure 9(a) and Figure 9(b). The actual lifetimes of tool No. 12 and tool No. 13 were measured to be 90.24 min and 97.63 min, respectively. Using the proposed operation reliability assessment method, the estimated lifetimes for tool No. 12 and tool No. 13 are 93.12 min and 96.41 min, respectively. Meanwhile, the estimated lifetimes of tool No. 12 and tool No. 13 are both 95.13 min, as determined by the conventional reliability assessment method. Table 3 shows a comparison between the two methods.

As illustrated in Figure 9 and Table 3, the estimation error of the proposed method is much smaller than that of the conventional reliability assessment method. Moreover, the result of the conventional method only reflects the population reliability of the identical units. The proposed method effectively reflects the characteristics of the cutting tool in operation by introducing the condition monitoring information of the cutting tool into the PCM.

**Figure 10.**
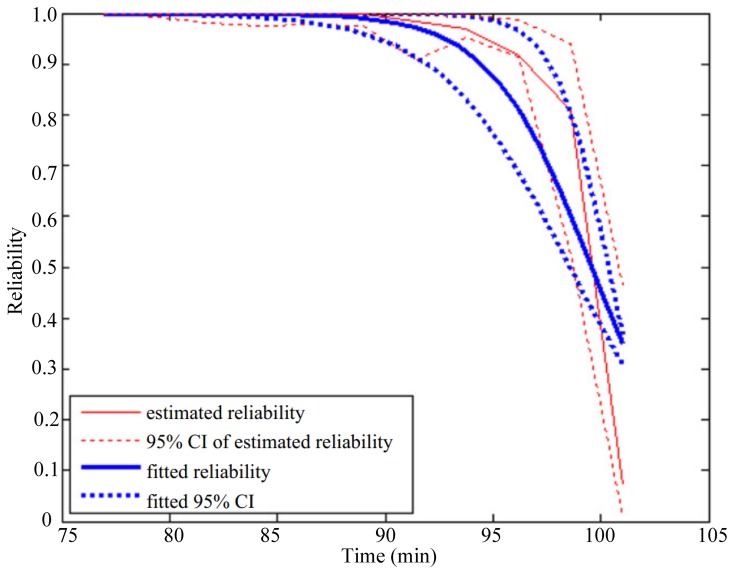
Reliability assessment result of the test tool determined by Chen's method [29].

To further validate the performance of the proposed method, the assessment result of the proposed method was compared with the assessment result of the previous study [29]. In [29], the reliability of the cutting tool was estimated using a logistic regression model based on vibration signals. The test cutting tool in [29] corresponds to tool No. 13 in the present study. Figure 10 shows the reliability assessment result of tool No.13 by using the logistic regression model introduced in [29].

It can be observed from Figure 10 that the estimated lifetime of tool No. 13 is 100 min. The estimated error is 1.42% in [29]. Meanwhile, using the method proposed in this study, the estimated lifetime of the tool is 96.41 min and the estimated error is 1.25%. The assessment result of the method proposed in this paper shows an improvement compared with the result presented in [29]. In addition, a 95% confidence interval (CI) is also given in Figure 10, as depicted by dashed lines.

### 4.5. Discussion

According to the results of the operation reliability assessment, it is convincing that by introducing the condition monitoring information of the cutting tool into reliability assessment, the proposed operation reliability assessment approach effectively reflects the characteristics of the cutting tool in operation. However, other issues still remain to be discussed.

**Figure 11.**
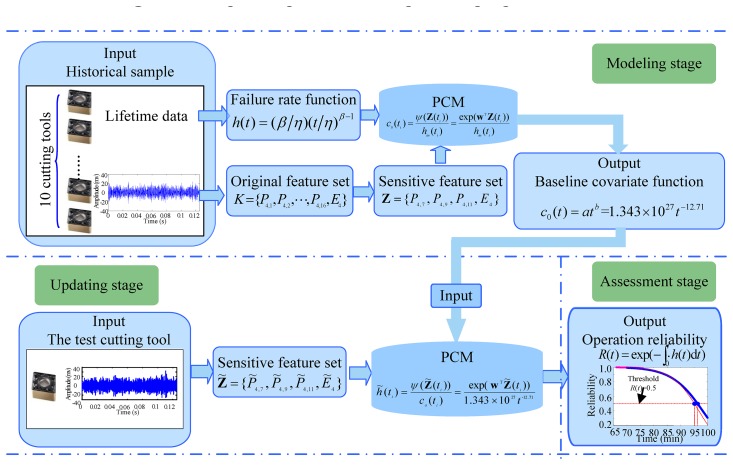
Input/output relationship of the proposed method.

1. The major contribution is that an operation reliability approach for assessing cutting tools by applying PCM and condition monitoring information is proposed. This contribution features two important aspects. First, by using PCM to introduce the condition monitoring information of a running cutting tool into operation reliability assessment, the method overcomes the main shortcoming of the conventional reliability assessment method: the inability to properly reflect the characteristic operation reliability of a given cutting tool. Second, PCM is introduced to assess operation reliability. In PCM, the baseline covariate function is employed to describe the relationship between condition monitoring information and operation reliability. The baseline covariate function represents the rate of change in the running condition when the operation reliability changes. Moreover, the baseline covariate function is dependent on both historical failure data and historical condition monitoring information; thus, it can be updated according to newly observed failure data and condition monitoring information.
2. The input, output, updating, and threshold setup of the model affect the performance of the proposed method. Figure 11 shows the fundamental input/output relationship of the proposed method.
  The input and output, as well as other factors that affect the estimation accuracy of the proposed method, are discussed within the context of each of the three stages in the method (the modeling stage, the updating stage and the assessment stage) as follows:
  - In the modeling stage, both the lifetime and vibration signals act as input information with which to build the assessment model. In the presented engineering experiment, 10 cutting tools' lifetime data and vibration signals acted as input to construct the PCM, where sample size scales with construction accuracy. However, when setting the parameter, there are two limitations that should be considered: (a) small sample size and (b) practical experiment or engineering practice. To construct the relationship between the vibration signals and operation reliability, the failure rate of the cutting tool estimated from the lifetime acted as the output initially. The ultimate goal of this stage is to obtain the baseline covariate function of the PCM. The baseline covariate function is the final output of this stage.
  - During the updating stage, the input is two-fold. First, there is the baseline covariate function, the output of the modeling stage. Then, there are the vibration signals of the cutting tool to be assessed. The output of this stage is the updated failure rate function of the cutting tool. During this stage, the sample of the input vibration signals is affected by practical experiment and computation effort.
  - During the assessment stage, what we are concerned about is the operation reliability threshold. In this paper, 0.5 was set to be the operation reliability threshold for the cutting tool.
  Moreover, the setup of the parameters mentioned above requires much more theoretical study and experimental work to provide more scientific rules.
3. In engineering applications, the measurement of flank wear is difficult because of the continuous contact between tools and workpieces, and it is fairly inconvenient due to the presence of coolant fluids. Thus, based on the present study, on-line condition monitoring information can be used to assess the operation reliability of the cutting tool being studied.
4. In this study, the effectiveness of the proposed method was verified by submitting the cutting tool under flank wear on a CNC lathe. However, this does not mean the proposed method is limited in assessing the reliability of a cutting tool under flank wear. The method is also applicable and can be properly generalised for the analysis of other types of degradation failure. It should be mentioned here that the proposed method is not suitable in situations when a sudden failure occurs during the machining process. Further research will be focused on the application of the proposed method to other types of degradation failure.

## 5. Conclusions

In this paper, an operation reliability assessment approach for cutting tools by applying PCM is proposed. Taking the condition monitoring information of the cutting tool that was analysed into consideration, the approach overcomes the main shortcoming of the conventional reliability assessment method: the inability to properly reflect the characteristics of a given cutting tool. WPT and an improved distance evaluation technology are employed to extract and select the relevant features that are most sensitive to the degradation of the cutting tool. The corresponding distance evaluation criteria of the sensitive features are adopted as their feature weights to construct the feature covariate function. Then, the PCM-based reliability assessment model can be constructed without specific knowledge about the degradation mechanism of the cutting tool.

The applications of the proposed approach in the operation reliability assessment of three cutting tools under flank wear on a CNC lathe confirmed that the proposed technique is effective in operation reliability assessment. Moreover, comparisons of the proposed method with the conventional method and another relatively new reliability assessment technique were made and testified the superiority of the proposed method. This study provides a foundation for developing specific production planning and tool management strategies to avoid unexpected downtime and economic loss.

In this study, information regarding wear values and that regarding other aspects of condition monitoring were fused to comprehensively assess a cutting tool's condition and corresponding machining performance. Based on reliability theory, PCM was used to construct the relationship between condition monitoring information and operation reliability and then evaluate the reliability of the cutting tool. Although the proposed method was used to evaluate the reliability of a cutting tool, this is not our ultimate goal. Further study should be undertaken to fuse other condition monitoring information about machining tools to assess the performance of equipment and machining quality.

## Figures and Tables

**Table 1.** Experimental conditions.

**CNC lathe**	**FTC-20**
Workpiece	45# Steel bars
Cutting tool	Type: Diamond carbide tool Model: CNMG120408-HM Material: 42CrMo4
Cutting conditions	Feed rate *f*: 0.15 mm/rev Cutting speed *v_c_*: 200 m/min Depth of cut *a_p_*: 2 mm

**Table 2.** Detailed information of the sensors.

**Sensor**	**Sensor model**	**Detailed information**
Acceleration sensor	PCB ICP352C34	◆ Sensitivity: 100 mv/g ◆ Working frequency range: 0.3 Hz–15 KHz ◆ Measurement range: ±50 g pk ◆ Resolution: 0.00015 g ◆ Temperature range: −54 °C to +93 °C ◆ Size: Φ50 mm × 160 mm ◆ Weight: 5.6 g
Optical microscope	MZDH0670	◆ Zoom objective magnification 0.58X∼7.0X ◆ Zoom radio 12:1 ◆ Working distance 82 mm (1× objective) ◆ Adjusting high-brightness long-life LED coaxial illumination ◆ The measurement to match between the support and the main body: Φ45 mm

**Table 3.** Reliability assessment results of the proposed method compared with those of the conventional method.

	**Real lifetime (min)**	**The conventional reliability assessment method**		**The proposed operation reliability assessment method**	

		**Estimated lifetime (min)**	**Error (%)**	**Estimated lifetime (min)**	**Error (%)**
Tool No. 11	96.83	95.13	1.76	96.33	0.52
Tool No. 12	90.24		5.42	93.12	3.19
Tool No. 13	97.63		2.56	96.41	1.25

## Abbreviations

**Acronyms**
CNC: Computerised Numerical Control
PCM: Proportional Covariate Model
WT: Wavelet Transform
WPT: Wavelet Packet Transform
MLE: Maximum Likelihood Estimation
CI: Confidence Interval

**Notations**
**Z**(*t*): Sensitive feature set
Z*_rm_*(*t*): *m*th feature in the sensitive feature set
*h*(*t*): Failure rate function
*c*_0_(*t*): Baseline covariate function
*h_in_*(*t*): Initial failure rate function
*h̃*(*t*): Updated failure rate function
*f*(*t*): Probability density function
*R*(*t*): Reliability function
*ψ*(**Z**(*t*)): Feature covariate function
*E_m_*: Wavelet energy entropy of the *m*th level decomposition
*X_(m,n)_*: *n*th frequency band coefficient of the *m*th level decomposition
*S_m,n_*: Single branch reconstruction of *X_(m,n)_*
*r_m,n_*: Amplitude of *S_m,n_*
*L*: Length of the original signal
*E_m,n_*: Wavelet packet energy of *S_m,n_*
*P_m,n_*: Normalised wavelet packet energy of *S_m,n_*
*K*: Original feature set
*J*: Feature number of each condition
*C*: Number of the conditions
*K_m,c,j_*: *j*th feature of the *m*th sample under the *c*th condition
$v_{j}^{\left(b\right)}$: Inter-class difference factor
*M_c_*: Sample number of the *c*th condition
*u_c,j_*: Average feature value of all samples under the same condition
*d_c,j_*: Average distance of the same condition samples
$d_{j}^{\left(w\right)}$: Average intra-class difference factor
$d_{j}^{\left(b\right)}$: Average distance between different sample conditions
$v_{j}^{\left(w\right)}$: Intra-class difference factor of $d_{j}^{\left(w\right)}$
*a_j_*: Distance evaluation criterion
*λ_j_*: Compensation factor
*ā_j_*: Normalised distance evaluation criterion
*ϕ*: Threshold for sensitive feature selection
*V_B_*: Flank wear value
*β*: Shape parameter of the Weibull distribution
*η*: Scale parameter of the Weibull distribution
*τ_n_*: Lifetime data
*m_f_*: Number of lifetime data
*M*: Dimension of the signal feature set
**w**: Feature weight vector
*F*(*t*): Failure distribution of the cutting tool
**Z̃**(*t*): Sensitive feature set of the cutting tool in operation
*Z̃_rm_*(*t*): *m*th sensitive feature of the cutting tool in operation
